# Bending and Elastic Vibration of a Novel Functionally Graded Polymer Nanocomposite Beam Reinforced by Graphene Nanoplatelets

**DOI:** 10.3390/nano9121690

**Published:** 2019-11-26

**Authors:** Yuewu Wang, Ke Xie, Tairan Fu, Congling Shi

**Affiliations:** 1 Key Laboratory for Thermal Science and Power Engineering of Ministry of Education, Beijing Key Laboratory of CO_2_ Utilization and Reduction Technology, Department of Energy and Power Engineering, Tsinghua University, Beijing 100084, China; wangyuewu@mail.tsinghua.edu.cn; 2 Institute of Systems Engineering, China Academy of Engineering Physics, Mianyang 621900, China; xkshake@163.com; 3 Beijing Key Lab of MFPTS, China Academy of Safety Science & Technology, Beijing 100029, China

**Keywords:** polymer-based nanocomposite, functionally graded material, graphene nanoplatelets, static analysis, free vibration, improved third order shear deformation theory, Chebyshev–Ritz method

## Abstract

A novel functionally graded (FG) polymer-based nanocomposite reinforced by graphene nanoplatelets is proposed based on a new distribution law, which is constructed by the error function and contains a gradient index. The variation of the gradient index can result in a continuous variation of the weight fraction of graphene nanoplatelets (GPLs), which forms a sandwich structure with graded mechanical properties. The modified Halpin–Tsai micromechanics model is used to evaluate the effective Young’s modulus of the novel functionally graded graphene nanoplatelets reinforced composites (FG-GPLRCs). The bending and elastic vibration behaviors of the novel nanocomposite beams are investigated. An improved third order shear deformation theory (TSDT), which is proven to have a higher accuracy, is implemented to derive the governing equations related to the bending and vibrations. The Chebyshev–Ritz method is applied to describe various boundary conditions of the beams. The bending displacement, stress state, and vibration frequency of the proposed FG polymer-based nanocomposite beams under uniformly distributed loads are provided in detail. The numerical results show that the proposed distributions of GPL nanofillers can lead to a more effective pattern of improving the mechanical properties of GPL-reinforced composites than the common ones.

## 1. Introduction

High-strength and multifunctional polymer-based nanocomposites with low-content carbon-based nanofillers have attracted widespread attentions due to their remarkable mechanical properties such as large deformation, stretchability, good chemical compatibility, and energy storage capacity [1,2,3,4,5,6,7,8,9,10]. That is because the carbon-based nanofillers, such as carbon nanotubes (CNTs), graphene nanoplatelets (GPLs), and graphene oxides (GOs), can provide unique nanostructures and superior properties in the nanocomposites. The functionally graded material (FGM) is a novel type of composite material whose mechanical properties smoothly and continuously vary in a preferred direction [11,12,13,14]. To better use the superior mechanical properties of carbon-based nanofillers and inspire from the concept of FGM, the functionally graded carbon nanotube-reinforced composite (FG-CNTRC) [15,16,17] and functionally graded graphene nanoplatelet reinforced composite (FG-GPLRC) [18,19,20] have been introduced, where the weight fractions of the CNTs and GPLs vary in the thickness direction.

The dispersion of nanofillers in the polymer matrix based on a reasonable graded distribution can effectively make use of reinforcements. To address the effects of nanofiller distributions on the mechanical behaviors of FG polymer-based nanocomposites, different types of distributions, such as the uniform distribution (UD), FG-V shape, FG-O shape and FG-X shape, were introduced and employed in many reports, e.g., references [21,22,23,24,25,26]. In addition, the distribution laws in forms of general polynomials have also been implemented [27,28,29]. All existing functions to describe the nanofiller distribution law are not adjustable since no adjustable parameter is included. Until now, the distribution law having adjustable parameters, which can lead to continuously graded mechanical properties for the nanofiller-reinforced polymer nanocomposites, has not been reported. It is believed that an adjustable distribution has great potential for introducing a novel type of nanocomposite, and also can be used to optimize the mechanical performances of the nanofiller-reinforced structures. The first contribution of the current work is to propose a new distribution law with an adjustable parameter as the gradient index.

In recent years, the graphene nanoplatelet (GPL) has been considered as an ideal reinforcement because of its excellent material properties, including high stiffness and strength. A polymer with a low concentration of GPLs exhibits distinct improvement in mechanical properties. Yang and his coauthors devoted great efforts to the bending, buckling, and vibration behaviors of FG-GPLRC structures [30,31,32]. Shen et al. [33,34] discussed various results of the nonlinear bending, vibration, and buckling behaviors of FG-GPLRC plates, panels, and shells. Mao and Zhang [35] examined the linear and nonlinear vibration responses of graphene-reinforced piezoelectric composite plates under external voltage excitation. Wang et al. [36] studied the vibration and bending behaviors of functionally graded nanocomposite doubly curved shallow shells reinforced by GPLs. Gholami et al. [37] investigated the nonlinear, harmonically excited vibration of a third-order shear deformable FG-GPLRC rectangular plate. Although the studies concerning on the mechanical behaviors of FG-GPLRC structures are massive, few studies focus on improving the distribution of the GPLs. Furthermore, the bending and vibration performances are the most basic and critical properties for structural components. Lezgy-Nazargah and Salahshuran [38] proposed a novel mixed-field theory with relatively low number of unknown variables for investigating bending and vibration analysis of multi-layered composite plates. Wattanasakulpong and Ungbhakorn [39] studied the bending, buckling and vibration behaviors of carbon-nanotube-reinforced composite (CNTRC) beams. Ghannadpour et al. [40] investigated the bending, buckling and vibration analyses of nonlocal Euler beams. Zhao et al. [41] analyzed the bending and vibrations of functionally graded trapezoidal nanocomposite plates reinforced with GPLs. The second contribution of our work is to introduce a novel FG-GPLRC beam using the proposed distribution law and investigate its bending and vibration behaviors.

In present work, a novel functionally graded graphene nanoplatelet reinforced composite (FG-GPLRC) is proposed by developing a new distribution law. The variation of the gradient index results in a continuous variation of the weight fraction of graphene nanoplatelets (GPLs) and forms graded mechanical properties within composite according to the thickness direction. The modified Halpin–Tsai micromechanics model is employed to evaluate the effective Young’s modulus. The bending and elastic vibration behaviors of the novel FG-GPLRC beams are investigated. An improved third-order shear deformation theory (TSDT), which is proved to have a higher accuracy, is implemented to derive the governing equations. Chebyshev–Ritz method is applied to describe various boundary conditions of the beam. Parametric studies are performed and present the bending displacement, stress state, and vibration frequency of the new FG-GPLRC beam under the uniformly distributed loads. The effects of gradient index of the proposed GPL distributions on the bending and vibration performance are addressed.

## 2. A Novel GPL-Reinforced Nanocomposite

### 2.1. Evaluation of Effective Mechanical Properties

It is well known that the Eshelby-Mori-Tanaka theory and the Halpin–Tsai micromechanics model are the most common methods to determine the effective elastic moduli of nanocomposite materials. Much literature suggest that the Eshelby-Mori-Tanaka theory is able to yield a more accurate prediction of the nanocomposite mechanical properties [42,43,44,45,46], and this conclusion was supported by experiments for CNT-reinforced nanocomposite.

The Halpin–Tsai model is based on the generalized self-consistent method. Although this model was initially developed for traditional fiber composites, successful efforts were undertaken to apply this model to nanofillers-reinforced nanocomposites by introducing orientation and shape factors that account for the geometry of the filler phase. The Halpin–Tsai model was used to calculate the effective Young’s modulus of a polymer nanocomposite reinforced by graphene nanoplatelets (GPLs), and the comparisons between the theoretical predictions and experimental results were also performed [47,48]. Due to the simple form in mathematics, the Halpin–Tsai micromechanics model has been widely employed to estimate the effective Young’s modulus of functionally graded graphene nanoplatelets reinforced composites [27,28,29,30,31,32,33,34,35,36,37]. The main objective of the current work is to propose an adjustable distribution law to find a more effective way to use the GPL reinforcements. It is believed that the effective material properties resulted from the Halpin–Tsai model can provide the results with some reference values. Thus, the Halpin–Tsai micromechanics model is selected.

A beam composed of FG-GPLRCs is considered herein. The length, width, and thickness of the beam are *L*, *b*, and *h*, respectively. The origin of the coordinate system is fixed at the center at the left end of the beam. The matrix of the nanocomposite is a polymer, and the GPL nanofillers are uniformly or non-uniformly dispersed across the thickness direction of the beam. A modified Halpin–Tsai model is used to calculate the effective Young’s modulus of the GPL/polymer composite. We assume that GPLs are effective rectangular solid fillers dispersed in a polymer matrix, and the effective Young’s modulus *E_C_* of the GPL/polymer composite is approximated by Voigt–Reuss model as follows [18]:

(1) $$E_{C}=\frac{3}{8}E_{L}+\frac{5}{8}E_{T}$$

where longitudinal modulus *E_L_* and transverse modulus *E_T_* are determined by the Halpin–Tsai model as follows:

(2a) $$E_{L}=\frac{1+\xi_{L}\eta_{L}V_{GPL}}{1-\eta_{L}V_{GPL}}E_{M}$$

(2b) $$E_{T}=\frac{1+\xi_{W}\eta_{W}V_{GPL}}{1-\eta_{W}V_{GPL}}E_{M}$$

Substituting Equations (2) and (3) into Equation (1), we obtain the following expression:

(3) $$E_{C}=\frac{3}{8}\frac{1+\xi_{L}\eta_{L}V_{GPL}}{1-\eta_{L}V_{GPL}}E_{M}+\frac{5}{8}\frac{1+\xi_{W}\eta_{W}V_{GPL}}{1-\eta_{W}V_{GPL}}E_{M}$$

where

(4a) $$\eta_{L}=\frac{\left(E_{GPL}/E_{M}\right)-1}{\left(E_{GPL}/E_{M}\right)+\xi_{L}}$$

(4b) $$\eta_{W}=\frac{\left(E_{GPL}/E_{M}\right)-1}{\left(E_{GPL}/E_{M}\right)+\xi_{W}}$$

where *E_M_* and *E_GPL_* are Young’s moduli of the polymer matrix and GPLs, respectively; *V_GPL_* is the volume fraction of the graphene nanoplatelets; *ξ_L_* and *ξ_W_* characterize the geometry and size of the GPL nanofillers, respectively, and are defined as follows:

(5a) $$\xi_{L}=2\left(\frac{l_{GPL}}{h_{GPL}}\right)$$

(5b) $$\xi_{W}=2\left(\frac{w_{GPL}}{h_{GPL}}\right)$$

where *l_GPL_*, *w_GPL_*, and *h_GPL_* are the average length, width, and thickness of GPLs, respectively. Mass density *ρ_C_* and Poisson’s ratio *v_C_* of the GPL/polymer nanocomposite are calculated by the rule of mixtures as follows:

(6a) $$\rho_{C}=V_{GPL}\rho_{GPL}+V_{M}\rho_{M}$$

(6b) $$v_{C}=V_{GPL}v_{GPL}+V_{M}v_{M}$$

where *V_M_* is the volume fraction of the polymer matrix; subscripts “*GPL*”, “*M*”, and “*C*” denote the GPLs, polymer matrix, and GPL/polymer nanocomposite, respectively. The volume fraction of GPLs is:

(7) $$V_{GPL}=\frac{g_{GPL}}{g_{GPL}+\left(\rho_{GPL}/\rho_{M}\right)\left(1-g_{GPL}\right)}$$

where *g_GPL_* is the total weight fraction of the GPLs in the nanocomposite.

### 2.2. A New GPL Distribution

Previous works proved that different reinforcement distributions significantly affect the mechanical properties of the composites. FG-X, FG-O and FG-V are the most common types of distributions for the GPLs or CNTs, where the total weight fractions or volume fractions of the GPLs or CNTs are constant regardless how the reinforcements dispersed in the polymer matrix.

The motivation of the present work is to find a GPL distribution with an adjustable parameter that controls the weight fraction of GPLs in a preferred direction and maintain a constant total weight fraction. This will be helpful to address the impacts of the GPL distributions on the structural responses and provide a new distribution for designing polymer nanocomposites and optimizing the mechanical properties of FG-GPLRC composites in different applications.

The new distribution function is constructed by using an error function:

(8) $$\left\{ \begin{array}{l} g_{GPL}\left(z\right)=g_{GPL}^{0}\frac{2r}{\sqrt{\pi }\mathrm{Erf}\left(r\right)}e^{-4r^{2}{\left(\frac{z}{h}-\frac{1}{2}\right)}^{2}}\;\;0\leq z\leq \frac{h}{2}\\ g_{GPL}\left(z\right)=g_{GPL}^{0}\frac{2r}{\sqrt{\pi }\mathrm{Erf}\left(r\right)}e^{-4r^{2}{\left(\frac{z}{h}+\frac{1}{2}\right)}^{2}}\;\;-\frac{h}{2}\leq z\leq 0 \end{array}\right.$$

where $\mathrm{Erf}\left(r\right)=\frac{2}{\sqrt{\pi }}\int_{0}^{r}e^{-t^{2}}dt$ is the error function; *r* is the gradient index and takes only positive values (*r* > 0); $g_{GPL}^{0}$ is the total weight fraction of the GPLs. Although the value of *r* cannot be zero, when the selected *r* approaches 0, the value of Equation (8) approaches 1 as its limit, which represents the uniform GPL distribution pattern. From Equation (8), the following features of the proposed GPL distribution are obtained:

1. The total weight fraction of GPLs $g_{GPL}^{0}$ remains constant with the *r* variations.
2. The GPLs symmetrically disperse in the matrix about the mid-plane of the beam.
3. When *r* increases, many GPLs are increasingly dispersed to the upper and lower surfaces of the beam.
4. This GPL graded distribution is continuous along the thickness and makes the mechanical properties of the FG-GPLRCs continuously vary.

**Figure 1:**
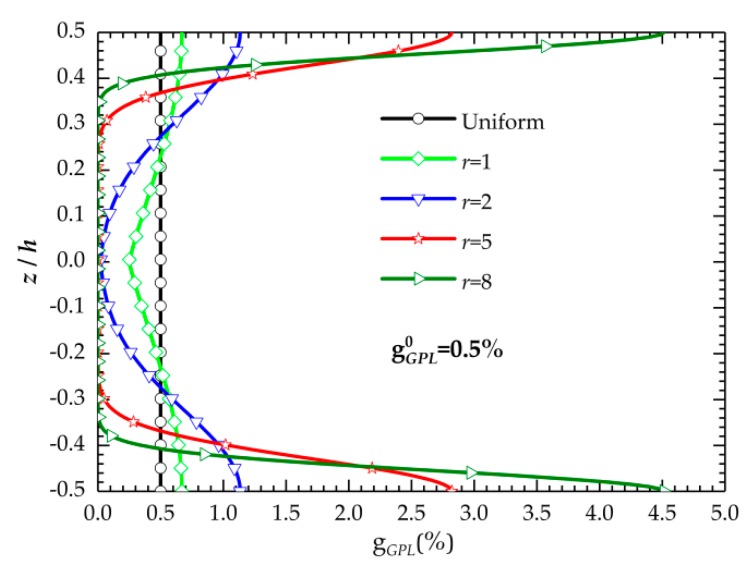
Variation of the weight fraction of the graphene nanoplatelets (GPLs) versus *r*.

To clearly illustrate the mentioned characteristics for the proposed distribution law, Figure 1 presents the variation of GPL weight fraction with respect to *r*. The total weight fraction of the GPLs in Figure 1 is assumed to be 0.5%.

**Figure 2:**
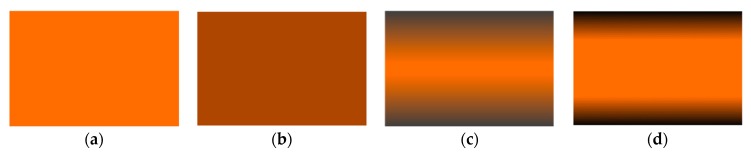
Schematic of GPL distributions of the proposed model: (**a**) without GPLs; (**b**) uniform distribution; (**c**) FG *r* = 2; (**d**) FG *r* = 5.

For further illustrating, Figure 2 presents the schematic of the GPL distributions of the proposed model with different values of the gradient index. The features of the new FG-GPLRCs are clearly observed.

In fact, a functionally graded GPL-reinforced composite (FG-GPLRC) structure is ideal in combining the advantages of both FGMs and GPLs. However, the fabrication of such functionally graded structures with a continuous and smooth variation of GPLs across the thickness is extremely difficult due to the constraint of manufacture technology. For overcoming this problem, the functionally graded GPL reinforced multilayer structures are introduced, because a functionally graded GPL reinforced multilayer nanocomposite structure in which each individual layer is made from a mixture of uniformly distributed GPL reinforcements and polymer matrix with GPL concentration incrementally varying layer by layer is much easier to fabricate. The published results have provided authentic evidence that such a multilayer structure is an excellent approximation to the ideal functionally graded structure with a continuous and smooth variation of GPLs across the thickness direction when the total number of layers is sufficiently large [18,31,32,49,50,51].

In the following, the dimensions of the graphene nanoplatelets are *l*_GPL_ = 2.5 µm, *w*_GPL_ = 1.5 µm, and *h*_GPL_ = 1.5 nm. The material properties of GPLs and epoxy are *ρ*_GPL_ = 1.06 g/cm^3^, *E*_GPL_ = 1.01 TPa, *ρ*_M_ = 1.2 g/cm^3^, *E*_M_ = 3.0 GPa, *v*_M_ = 0.34, and *v*_GPL_ = 0.186.

## 3. Theory and Formulations

In the present work, an accurate and efficient modeling is established based on an improved third-order shear deformation theory, and the governing equations associated with the static and vibration problems of the beams with various boundary conditions are derived using the minimum energy methodology and Chebyshev–Ritz method.

### 3.1. An Improved Third Order Shear Deformation Theory

The improved third-order shear deformation theory (TSDT) was originally proposed by Shi [52] based on rigorous kinematic of displacements. The published literature has proven that the results obtained by the improved TSDT are more reliable and accurate than many other theories because the kinematics of displacements is derived from elasticity theory instead of the hypothesis of displacement like other existing approaches.

The displacement fields of the improved third-order shear deformation theory (TSDT) are expressed as follows [52,53]:

(9) $$\left\{ \begin{array}{l} u=u_{0}\left(x,t\right)+\frac{5}{4}\left(z-\frac{4}{3h^{2}}z^{3}\right)\phi_{x}\left(x,t\right)+\left(\frac{1}{4}z-\frac{5}{3h^{2}}z^{3}\right)\frac{\partial w_{0}(x,t)}{\partial x}\\ w=w_{0}\left(x,t\right) \end{array}\right.$$

where *u*_0_ and *w*_0_ define the generalized displacements at the mid-plane of the beam in the *x* and *z* directions, and *φ_x_* is the rotation of the beam. From the above displacement fields, the small normal strain *ε_xx_* and transverse shear strain *γ_xz_* can be written as

(10) $$\left\{ \begin{array}{l} \varepsilon_{xx}=\frac{\partial u}{\partial x}=\frac{\partial u_{0}}{\partial x}+\frac{z}{4}\left(5\frac{\partial \phi_{x}}{\partial x}+\frac{\partial^{2}w_{0}}{\partial x^{2}}\right)-\frac{5z^{3}}{3h^{2}}\left(\frac{\partial \phi_{x}}{\partial x}+\frac{\partial^{2}w_{0}}{\partial x^{2}}\right)\\ \gamma_{xz}=\frac{\partial u}{\partial z}+\frac{\partial w}{\partial x}=\frac{5}{4}\left(\phi_{x}+\frac{\partial w_{0}}{\partial x}\right)-\frac{5z^{2}}{h^{2}}\left(\phi_{x}+\frac{\partial w_{0}}{\partial x}\right) \end{array}\right.$$

Then, the stresses are obtained under the assumption of Hook’s law:

(11) $$\left\{ \begin{array}{l} \sigma_{xx}=Q_{11}\left(z\right)\varepsilon_{xx}\\ \sigma_{xz}=Q_{55}\left(z\right)\varepsilon_{xz} \end{array}\right.$$

where *Q*_11_(*z*) and *Q*_55_(*z*) are the elastic constants that continuously vary along the beam thickness and expressed as

(12) $$\left\{ \begin{array}{l} Q_{11}(z)=\frac{E\left(z\right)}{1-\nu^{2}}\\ Q_{55}\left(z\right)=\frac{E\left(z\right)}{2\left(1+\nu \right)} \end{array}\right.$$

The strain energy *U* of the beam is

(13) $$U=\frac{1}{2}\int_{0}^{L}\int_{-h/2}^{h/2}b\left(\sigma_{xx}\varepsilon_{xx}+\sigma_{xz}\varepsilon_{xz}\right)dzdx$$

Substituting Equations (9)–(12) into Equation (13), we obtain the strain energy expression as a function of the material stiffness and strain components as

(14) $$U=\frac{1}{2}\int_{0}^{L}\left\{\begin{array}{l} A_{11}{\left(\frac{\partial u_{0}}{\partial x}\right)}^{2}+\frac{B_{11}}{2}\left(5\frac{\partial u_{0}}{\partial x}\frac{\partial \phi_{x}}{\partial x}+\frac{\partial u_{0}}{\partial x}\frac{\partial^{2}w_{0}}{\partial x^{2}}\right)+\frac{D_{11}}{16}{\left(5\frac{\partial \phi_{x}}{\partial x}+\frac{\partial^{2}w_{0}}{\partial x^{2}}\right)}^{2}\\ -\frac{10E_{11}}{3h^{2}}\left(\frac{\partial u_{0}}{\partial x}\frac{\partial \phi_{x}}{\partial x}+\frac{\partial u_{0}}{\partial x}\frac{\partial^{2}w_{0}}{\partial x^{2}}\right)-\frac{5F_{11}}{6h^{2}}\left[5{\left(\frac{\partial \phi_{x}}{\partial x}\right)}^{2}+6\frac{\partial \phi_{x}}{\partial x}\frac{\partial^{2}w_{0}}{\partial x^{2}}+{\left(\frac{\partial^{2}w_{0}}{\partial x^{2}}\right)}^{2}\right]\\ +\frac{25}{9h^{4}}H_{11}{\left(\frac{\partial \phi_{x}}{\partial x}+\frac{\partial^{2}w_{0}}{\partial x^{2}}\right)}^{2}+\frac{25}{16}A_{55}{\left(\phi_{x}+\frac{\partial w_{0}}{\partial x}\right)}^{2}-D_{55}\frac{25}{2h^{2}}{\left(\phi_{x}+\frac{\partial w_{0}}{\partial x}\right)}^{2}+\frac{25}{h^{4}}F_{55}{\left(\phi_{x}+\frac{\partial w_{0}}{\partial x}\right)}^{2} \end{array}\right\}dx$$

where *A*_11_, *B*_11_, *D*_11_, *F*_11_, *H*_11_, *A*_55_, *D*_55_ and *F*_55_ are the stiffness constants, and they are defined as

(15) $$\left\{ \begin{array}{l} \left(A_{11},B_{11},D_{11},E_{11},F_{11},H_{11}\right)=b\int_{-h/2}^{h/2}Q_{11}\left(z\right)\left(1,z,z^{2},z^{3},z^{4},z^{6}\right)dz\\ \left(A_{55},D_{55},F_{55}\right)=b\int_{-h/2}^{h/2}Q_{55}\left(z\right)\left(1,z^{2},z^{4}\right)dz \end{array}\right.$$

The kinetic energy of the composite beam is

(16) $$T=\frac{1}{2}\int_{0}^{L}\int_{-h/2}^{h/2}b\left\{\rho \left(z\right)\left[{\left(\frac{\partial u}{\partial t}\right)}^{2}+{\left(\frac{\partial w}{\partial t}\right)}^{2}\right]\right\}dzdx$$

where *ρ*(*z*) is the mass density of the beam, which varies in the thickness direction.

Substituting Equation (9) into Equation (16), we can rewrite the kinetic energy as

(17) $$T=\frac{1}{2}\int_{0}^{L}\left\{\begin{array}{l} I_{0}\left[{\left(\frac{\partial u_{0}}{\partial t}\right)}^{2}+{\left(\frac{\partial w_{0}}{\partial t}\right)}^{2}\right]+\frac{I_{1}}{2}\left(5\frac{\partial u_{0}}{\partial t}\frac{\partial \phi_{x}}{\partial t}+\frac{\partial u_{0}}{\partial t}\frac{\partial^{2}w_{0}}{\partial x\partial t}\right)+\frac{I_{2}}{16}\left[25{\left(\frac{\partial \phi_{x}}{\partial t}\right)}^{2}+10\frac{\partial \phi_{x}}{\partial t}\frac{\partial^{2}w_{0}}{\partial x\partial t}+{\left(\frac{\partial^{2}w_{0}}{\partial x\partial t}\right)}^{2}\right]\\ -\frac{10I_{3}}{3h^{2}}\left(\frac{\partial u_{0}}{\partial t}\frac{\partial \phi_{x}}{\partial t}+\frac{\partial u_{0}}{\partial t}\frac{\partial^{2}w_{0}}{\partial x\partial t}\right)-\frac{5I_{4}}{6h^{2}}\left[5{\left(\frac{\partial \phi_{x}}{\partial t}\right)}^{2}+6\frac{\partial \phi_{x}}{\partial t}\frac{\partial^{2}w_{0}}{\partial x\partial t}+{\left(\frac{\partial^{2}w_{0}}{\partial x\partial t}\right)}^{2}\right]\\ +\frac{25I_{6}}{9h^{4}}\left[{\left(\frac{\partial \phi_{x}}{\partial t}\right)}^{2}+2\frac{\partial \phi_{x}}{\partial t}\frac{\partial^{2}w_{0}}{\partial x\partial t}+{\left(\frac{\partial^{2}w_{0}}{\partial x\partial t}\right)}^{2}\right] \end{array}\right\}dx$$

where $I_{i}=b\int_{-h/2}^{h/2}\rho (z)z^{i},\;i=0,1,2,3,4,6$ is the inertia terms.

The work done by a uniform distributed load *q* is:

(18) $$V=-\int_{0}^{L}qw\left(x\right)dx$$

The total energy function (Π) of the FG-GPLRC beam for the bending problem can be written as:

(19) Π=U+V

The total energy functional (Π) of FG-GPLRC beams for the free-vibration analysis is expressed as:

(20) Π=U−T

### 3.2. Chebyshev–Ritz Method

Ritz method is known as an effective tool to analyze the structural behavior. Since the adoption of trial functions only depends on the essential type of boundary condition [54], various functions may be selected as the admissible functions. In the present work, each displacement function can be written in the form of triplicate series of Chebyshev polynomials multiplied by a boundary function, which ensures that the displacement component satisfies the essential geometric boundary condition of the beam, i.e.,

(21) $$\left\{ \begin{array}{l} u_{0}\left(x,t\right)=B_{u}\left(x\right)\sum_{i=1}^{N}{\bar{U}}_{i}\left(t\right)P_{i}\left(x\right)\\ w_{0}\left(x,t\right)=B_{w}\left(x\right)\sum_{i=1}^{N}{\bar{W}}_{i}\left(t\right)P_{i}\left(x\right)\\ \phi_{x}\left(x,t\right)=B_{\varphi }\left(x\right)\sum_{i=1}^{N}{\bar{V}}_{i}\left(t\right)P_{i}\left(x\right) \end{array}\right.$$

where *N* is the truncated number of Chebyshev polynomials. *B*_Ξ_ (*x*) (Ξ = *u*, *w* and *ϕ*) are the boundary functions; $\left({\bar{U}}_{n},{\bar{W}}_{n},{\bar{V}}_{n}\right)$ denote the unknown coefficients corresponding to time and are expressed as follows: $\left({\bar{U}}_{n},{\bar{W}}_{n},{\bar{V}}_{n}\right)=\left(U_{n},W_{n},V_{n}\right)e^{i\omega_{n}t}$, $i=\sqrt{-1}$, where *ω_n_* is the vibration frequency.

*P_i_*(*x*) in Equation (21) is the *i*-th Chebyshev polynomial of the first kind, which is commonly known as “the most optimal expansion” [55,56], and defined in the interval of [−1, 1] as:

(22) $$P_{i}\left(x\right)=\cos\left(\left(i-1\right)\arccos\left(\frac{2x}{L}-1\right)\right),\;i=1,2,3\ldots$$

The recursive relationship is

(23) $$\left\{ \begin{array}{l} P_{0}\left(\chi \right)=1\\ P_{1}\left(\chi \right)=\chi \\ P_{i+1}\left(\chi \right)=2\chi P_{i}\left(\chi \right)-P_{i-1}\left(\chi \right) \end{array}\right.$$

There are two distinct advantages of selecting Chebyshev polynomial series as the admissible functions for each displacement component [57,58]: (1) *P_i_*(*x*) is a set of complete and orthogonal series in the interval of [−1, 1] and has more rapid convergence and better numerical stability in computation than other polynomials; (2) *P_i_*(*x*) can be expressed in a simple and unified form of cosine function as shown in Equation (22), which reduces the coding efforts.

The boundary functions *B*_Ξ_(*x*) (Ξ = *u*, *w* and *ϕ*) that correspond to *u*, *w* and *ϕ* are provided in the following uniform formula:

(24) $$B_{\Theta }\left(x\right)={\left(\frac{x}{L}\right)}^{L_{\Theta }}{\left(1-\frac{x}{L}\right)}^{R_{\Theta }}$$

where *L*_Θ_ and *R*_Θ_ are indices from the following essential geometric boundary conditions:

(1) Hinged-Hinged (H-H)
  *x* = 0: *u*_0_ = 0; *w* = 0; *φ_x_* ≠ 0
  *x* = *L*: *u*_0_ = 0; *w* = 0; *φ_x_* ≠ 0
(2) Clamped-Clamped (C-C)
  *x* = 0: *u*_0_ = 0; *w* = 0; $\frac{dw}{dx}=0$; *φ_x_* ≠ 0
  *x* = *L*: *u*_0_ = 0; *w* = 0; $\frac{dw}{dx}=0$; *φ_x_* ≠ 0
(3) Clamped-Hinged (C-H)
  *x* = 0: *u*_0_ = 0; *w* = 0; $\frac{dw}{dx}=0$; *φ_x_* = 0
  *x* = *L*: *u*_0_ = 0; *w* = 0; *φ_x_* = 0

Table 1 lists the values of the indices for various boundary conditions.

Substituting Equation (21) into the total energy functional (Π) for the bending in Equation (19) and free vibration in Equation (20) and taking the derivative with respect to the unknown coefficients in the procedure of finding minimization requires

(25) $$\frac{\partial \Pi }{\partial U_{n}}=0,\frac{\partial \Pi }{\partial W_{n}}=0,\frac{\partial \Pi }{\partial V_{n}}=0$$

The aforementioned procedure produces a system of simultaneous equations with an equal number of unknown coefficients (*U_n_*, *W_n_*, *V_n_*).

The equation system of solving the static bending problem of the FG-GPLRC beam under a distributed load *q* can be given as:

(26) $$\left[K\right]\bar{\Delta }=F$$

where [**K**] is the structural stiffness matrix; **F** is a column vector associated with the external load from Equation (17); and $\bar{\Delta }$ is the solution column vector for the static problem.

The generalized eigenvalue problem for free vibration is expressed as follows:

(27) $$\left(\left[K\right]-\omega^{2}\left[M\right]\right)\Delta =0$$

where [**K**] is the structural stiffness matrix, [**M**] is the mass matrix, and *ω* is the natural frequency. Vector Δ is the eigenvector from the displacement functions, which represents the modal shapes of the structures. The dimensions of the aforementioned matrices are 3 *N* × 3 *N*. The dimension of Δ is 3 *N* × 1.

## 4. Convergence and Validation Studies

The validation and accuracy of the current model developed based on the improved third-order shear deformation theory and Chebyshev–Ritz method are verified by comparing the transverse deflections, stresses and vibration frequencies of the FG and FG sandwich beams reported in the existing literature.

A hinged-hinged (H-H) sandwich beam with power-law type FG face sheets and a homogeneous hardcore, which is subjected to a uniform distributed load, is selected for the first example. The power-law index of the facesheets is 0.5. The top and bottom surfaces of the beam are metal-rich, while its core is made of ceramic. The material properties of the metal (Al) are *E* = 70 GPa and *ρ* = 2702 kg/m^3^, and those of ceramic (Al_2_O_3_) are *E* = 380 GPa and *ρ* = 3960 kg/m^3^. Both Poisson’s ratios are 0.3. The length-to-height ratio of the sandwich beam is set to be 5 (*L*/*h* = 5), and its layer thickness ratio is 2:1:2.

The transverse deflection (*w*) at mid-span, normal stresses (*σ_xx_*) at the top surface of the mid-span, transverse shear stress (*σ_xz_*) at mid-plane at the left end, and natural frequencies of the sandwich beam are calculated and compared with the results from [59] in the following dimensionless form:

$$\bar{w}=\frac{wh^{3}}{12}\frac{384E_{m}}{5q_{0}L^{4}},{\bar{\sigma }}_{xx}=\sigma_{xx}(\frac{L}{2},z)\frac{h}{q_{0}L},{\bar{\sigma }}_{xz}=\sigma_{xz}(0,z)\frac{h}{q_{0}L},\omega_{i}=\frac{\Omega_{i}L^{2}}{h}\sqrt{\frac{\rho_{m}}{E_{m}}}$$

Table 2 tabulates the dimensionless results, which show excellent consistency between the present results and the published results based on a high-order shear deformation theory. Meanwhile, 6 is a reasonable number of Chebyshev polynomial terms, which is sufficiently large to obtain accurate results.

In addition, Table 3 and Table 4 list the dimensionless mid-span displacements and fundamental frequencies of the sandwich beams with the layer thickness ratio of 1:2:1. The H-H and C-C boundary conditions are used, and various power-law indices (*p*) are considered. The results are compared with the available ones obtained by the first-order shear deformation theory (FSDT), third-order shear deformation theory (TSDT) and quasi-3D beam theory from the literature [60,61]. The present results well match the published results. It is important to note that the numerical results from the present model are closer to the results from the quasi-3D theory than the other beam theories, which indicates that the present model is accurate and efficient.

## 5. Results and Discussion

To facilitate the presentation, the following dimensionless parameters are introduced as follows:

$$\mathrm{Vertical}\;\mathrm{displacement}:\;\bar{w}\left(x\right)=w\left(x\right)\frac{100E_{M}h^{3}}{qL^{4}}$$

$$\mathrm{Dimensionless}\;\mathrm{normal}\;\mathrm{stress}:\;{\bar{\sigma }}_{xx}=\sigma_{xx}\left(\frac{L}{2},z\right)\frac{h}{qL}$$

$$\mathrm{Dimensionless}\;\mathrm{shear}\;\mathrm{stress}:\;{\bar{\sigma }}_{xz}=\sigma_{xz}\left(0,z\right)\frac{h}{qL}$$

$$\mathrm{Dimensionless}\;\mathrm{frequencies}:\;\Omega_{i}=\frac{\omega_{i}L^{2}}{h}\sqrt{\frac{\rho_{M}}{E_{M}}}$$

where *ρ*_M_ and *E*_M_ are the density and elastic modulus of the polymer matrix, respectively.

### 5.1. Bending Analysis

#### 5.1.1. Bending Deflection

In this section, the effects of the gradient indices and GPL weight fractions on the bending behaviors of the new FG-GPLRC beams are investigated. Table 5, Table 6 and Table 7 present the numerical results of the maximum dimensionless displacement ${\bar{w}}_{\max}$ of the FG-GPLRC beams with various boundary conditions.

**Figure 3:**
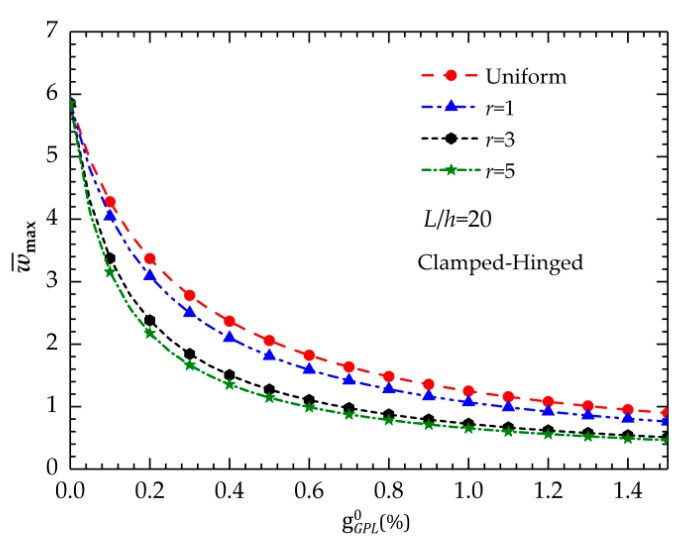
Variation in the maximum dimensionless displacement ${\bar{w}}_{\max}$ with respect to the GPL weight fraction $g_{GPL}^{0}$.

Figure 3 shows the relationship between maximum dimensionless displacement ${\bar{w}}_{\max}$ and GPL weight fractions. There is a nonlinear variation between the values of ${\bar{w}}_{\max}$ with respect to the GPL weight fractions. Regardless of the gradient index, ${\bar{w}}_{\max}$ decreases when the total weight fraction of the GPLs $g_{GPL}^{0}$ increases. The curves in Figure 3 clearly indicate that the decrease in ${\bar{w}}_{\max}$ increases when the GPL content is relatively low, while the decrease tends to be slight when the GPL content increases to a relatively high level, which suggests that the bending response of the beam is more sensitive to the variations of the GPL contents when $g_{GPL}^{0}$ is relatively low.

**Figure 4:**
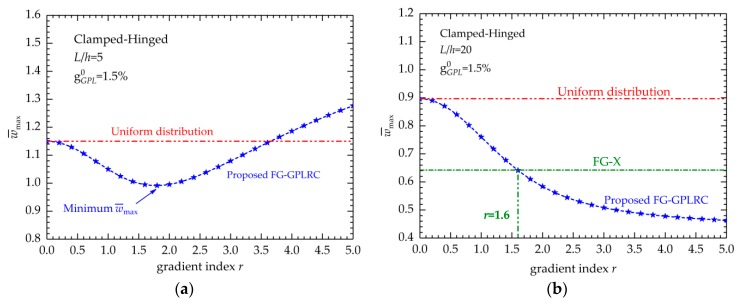
Relationship between maximum dimensionless displacement ${\bar{w}}_{\max}$ and gradient index *r*: (**a**) *L*/*h* = 5; (**b**) *L*/*h* = 20.

Another concerning point is the effect of the gradient index on the bending displacements of the beam. Figure 4 presents the relationship between the maximum dimensionless displacements ${\bar{w}}_{\max}$ and the values of gradient index *r*. *L*/*h* = 5 and *L*/*h* = 20 are considered. The curves in Figure 4 indicate that parameter *r* has remarkable effects on the bending performances of the FG-GPLRC beam because the variation of *r* changes the variation of GPLs in the thickness direction of the beam when the weight fraction is fixed and affects the beam stiffness.

For the thick beam (*L*/*h* = 5), as shown in Figure 4a, the regulation between *r* and ${\bar{w}}_{\max}$ becomes complex and interesting. When the total weight fraction $g_{GPL}^{0}$ is very small, e.g., 0.25%, the increase in *r* will result in a decrease of ${\bar{w}}_{\max}$ implies that dispersing more reinforcements near the surfaces can improve the bending resistance of the beam. However, when the total weight fraction $g_{GPL}^{0}$ is relatively high, e.g., 1.25%, when gradient index *r* increases, ${\bar{w}}_{\max}$ first reduces and subsequently increases, which suggests that there is a gradient index value that can minimize ${\bar{w}}_{\max}$. This *r* value can produce the best GPL distribution that provides the greatest improvement to the FG-GPLRC beam.

For the thin beam (*L*/*h* = 20), as shown in Figure 4b, the maximum dimensionless displacements ${\bar{w}}_{\max}$ decrease when gradient index *r* increases regardless of the geometric parameters and boundary conditions. As mentioned, when the total GPL weight fractions remain constant, the increase in *r* implies that more GPLs are dispersed in the top and bottom surfaces of the beam. Therefore, Figure 4b indicates that dispersing more GPL nanofillers near the top and bottom surfaces of the beam is most effective in enhancing the beam stiffness. The numerical results in Table 5, Table 6 and Table 7 also support this conclusion.

In addition, for the thin beam, Figure 4b demonstrates that there is a nonlinear descent in the maximum dimensionless displacements ${\bar{w}}_{\max}$ versus the increase in gradient index *r*. For comparison, the maximum dimensionless displacement ${\bar{w}}_{\max}$ of the polymer composite beam of dispersing the GPLs in a FG-X type and UD type, are also displayed in Figure 4b. Figure 4b shows an intersection point of the two curves, which implies that the proposed FG-GPLRC beam and conventional FG-X composite beams have identical bending deflections. The corresponding value of *r* for this point will be of significance because when *r* is beyond this point, the new FG-GPLRC beams can provide better deformed-resistant capability than the conventional FG-X ones. In addition, we find that the value in Figure 4b is equal to 1.6 (*r* = 1.6), and this *r* at the intersection point is constant regardless of the boundary conditions, geometric parameters and total GPL weight fractions. This result can be mathematically explained that when *r* = 1.6, the values of the function from Equation (8) are closer to those from the FG-X function ($g\left(z\right)=4\left|\frac{z}{h}\right|$) at a given z-position, i.e., the GPL distribution along the thickness direction is similar to the GPL distribution from the FG-X one when *r* = 1.6.

#### 5.1.2. Stress State 

A hinged-hinged FG-GPLRC beam with a length-to-height ratio of 5 is used to evaluate the normal and shear stresses in the thickness direction, which reveals the effects of the gradient index and GPL weight fraction on the stress distributions of the beam.

**Figure 5:**
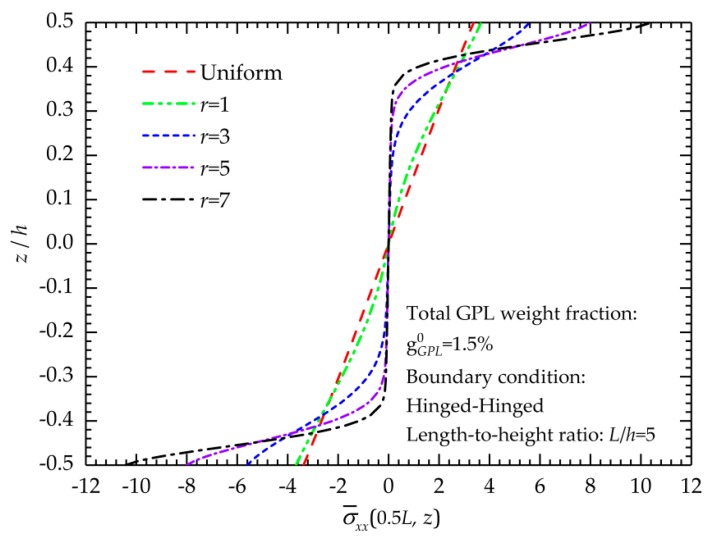
Normal stress distributions of the FG-GPLRC beam with different *r* values.

The effect of the gradient index on the normal stress distributions is displayed in Figure 5. Figure 5 shows that the normal stress linearly varies with the thickness coordinates when *r* approaches 0 because in this situation, the GPLs are uniformly dispersed in the polymer matrix, and the mechanical properties of the FG-GPLRCs have no change in the thickness direction. When *r* increases, nonlinear change occurs and become more remarkable because a greater *r* indicates that more GPLs are concentrated on the top and bottom surfaces, which makes a significantly uneven GPL distribution and produces the graded mechanical properties of the FG-GPLRCs. In addition, the increasingly concentrated dispersion of GPLs to the surfaces increases the normal stresses near the top and bottom of the beam when the gradient index increases but reduces those in the middle part of the beam. As mentioned, when *r* increases, the new FG-GPLRC beam can become a sandwich structure, and the stress distributions in Figure 5 prove the presence of the smooth and continuous variation of stresses throughout the entire cross-section.

**Figure 6:**
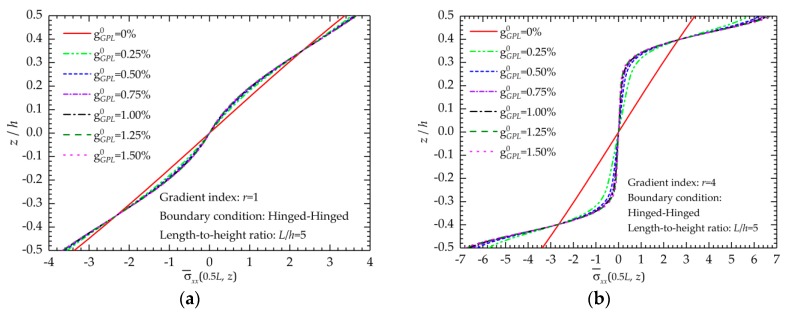
Effects of the total weight fractions of GPLs on the normal stress distributions: (**a**) *r* = 1; (**b**) *r* = 4.

Meanwhile, the effects of the total weight fractions of the GPLs on the normal stress distributions are presented in Figure 5. The cases of *r* = 1 and *r* = 4 are considered in Figure 6a,b, respectively. The variations of normal stresses are linear when $g_{GPL}^{0}=0$ for a pure polymer beam, but they nonlinearly change when $g_{GPL}^{0}>0$ because the uneven distribution of GPLs produces the gradient variation of material properties. Further addition of GPLs increases the normal stresses near the top and bottom surfaces of the beam and reduces those in the internal part far away from the two surfaces.

**Figure 7:**
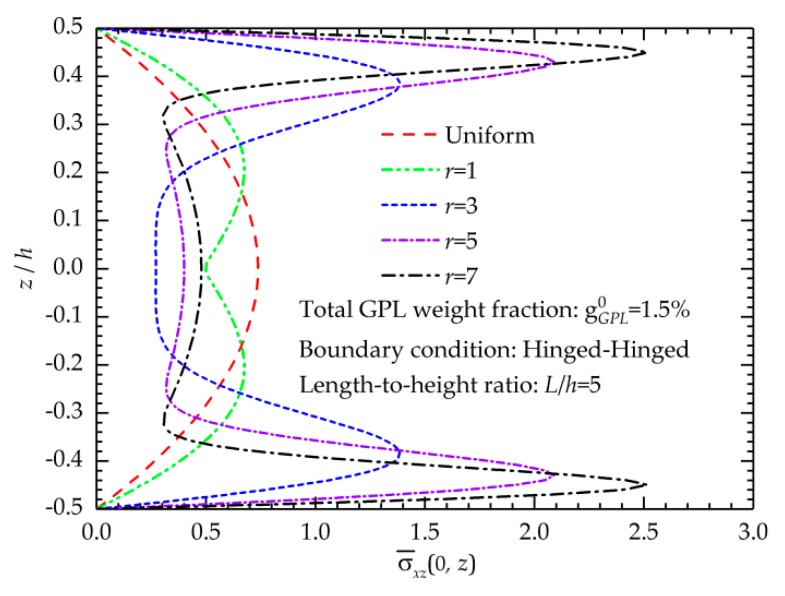
Effects of the gradient index on the shear stress distributions of the FG-GPLRC beam.

Figure 7 shows the variation of the shear stress distributions in the thickness direction of the FG-GPLRC beams with various gradient index values. To satisfy the traction-free condition on the surfaces of the improved TSDT, the shear stresses on the top and bottom surfaces are zero, and the maximum shear stress appears near the mid-surface of the beam. The curves in Figure 7 show that the change in gradient index significantly affects the shear stress distribution. When the gradient index increases, the maximum shear stress moves to the beam surfaces.

**Figure 8:**
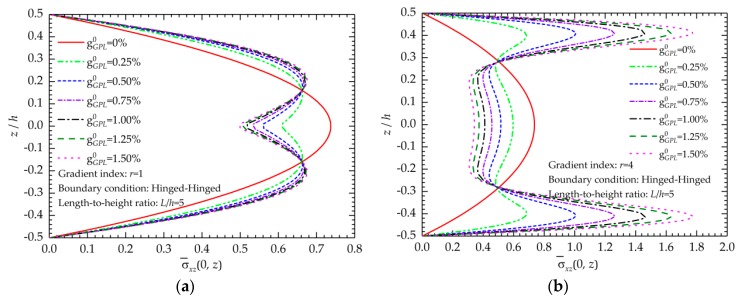
Effects of the total weight fractions of GPLs on the shear stress distribution: (**a**) *r* = 1; (**b**) *r* = 4.

Figure 8 shows the effects of the total weight fraction on the shear stress distributions. As observed in Figure 8, when the gradient index is fixed, the increase in GPL weight fraction increases the shear stresses near the top and bottom surfaces and reduces those in the center portion of the beam.

### 5.2. Elastic Vibration Behavior

Table 8, Table 9 and Table 10 show the dimensionless fundamental frequencies of FG-GPLRC beams with different gradient indices and weight fractions. The total weight fraction of the GPL is 0–1.5% with an increment of 0.25%. Three immovable boundary conditions are considered.

The numerical results in Table 8, Table 9 and Table 10 indicate that the change in gradient index can significantly affect the fundamental frequency and consequently affect the elastic vibration characteristics of the composite beam. The effect becomes increasingly obvious when the total weight fraction increases. For a fixed concentration of GPLs in the FG-GPLRC beam, when the gradient index increases, the dimensionless frequency increases. As mentioned, the increase in gradient index implies that more GPLs are dispersed near the top and bottom surfaces of the beam. Therefore, the above phenomenon proves that dispersing more GPL nanofillers near the top and bottom surfaces of the beam is most effective in enhancing the beam stiffness.

**Figure 9:**
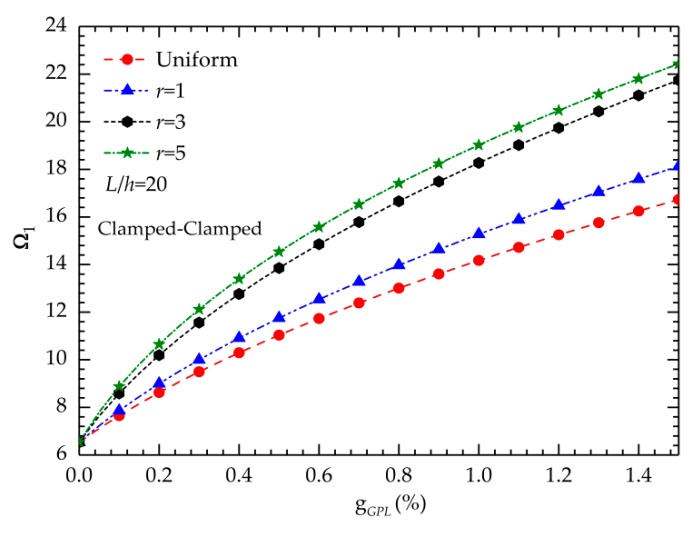
Variation of the dimensionless fundamental frequency with respect to the gradient index.

The curves in Figure 9 reflect the relationship between the fundamental frequencies and the weight fractions. The results in Figure 9 indicate that the fundamental frequencies increase when the GPL concentration increases regardless of the gradient index. The aforementioned phenomenon clearly indicates that low-content GPL additions significantly improve the stiffness of the beam.

**Figure 10:**
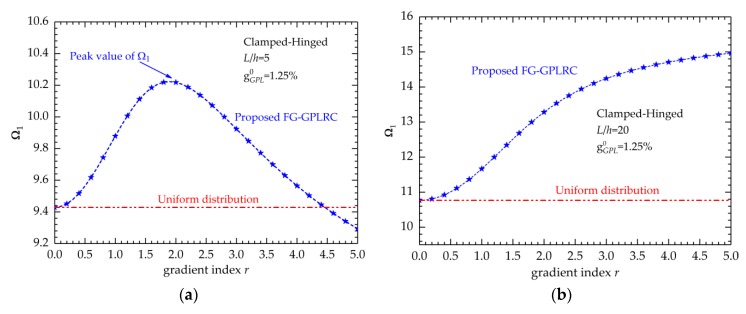
Variations of the maximum dimensionless displacements with respect to gradient index *r*: (**a**) *L*/*h* = 5; (**b**) *L*/*h* = 20.

Figure 10 shows the change in dimensionless fundamental frequencies with respect to the gradient index. The total weight fraction of GPLs is 1.25%.

Figure 10a shows the results of the thick beam (*L*/*h* = 5), and Figure 10b is related to the thin one (*L*/*h* = 20). We can reach a contrary conclusion with that of the bending behaviors: for the thick beam, when the gradient index increases, the fundamental frequencies first increase and subsequently decrease. There is a suitable *r* value that can result in a peak fundamental frequency, which indicates that the FG-GPLRC beam with this *r* value is the stiffest. However, this value is not constant and related to the geometric, weight fraction and boundary conditions.

For the thin beam (*L*/*h* = 20), the fundamental frequency increases with the increase in gradient index. A remarkable variation is observed when *r* is relatively small, and the variation becomes slight when *r* increases. This result demonstrates that the frequency is more sensitive to the gradient index when the gradient index is relatively small.

As mentioned before, the multilayer structures are introduced to solve the manufacturing problems caused by the distribution laws in practice. It is expected that as a total layer number increase, the functionally graded graphene reinforced multilayer beam will have the some static and vibration response to the monolithic FG-GPLRC beams. Therefore, we calculated the static displacements and fundamental frequencies of the multilayered nanocomposite beams with various layer numbers and made a comparison with those from the monolithic ones. It is worth to note that the weight fractions of each layer GPL-reinforced nanocomposites are determined by substituting the corresponding z-coordinate of mid-plane of each layer into Equation (8). In this example, the length-to-height ratio is set to be 5, and the total weight fraction of GPLs is 1.0%. The clamped-clamped boundary condition is taken into account. Table 11 lists the maximum displacements and fundamental frequencies of GPL-reinforced multilayer beams for different gradient index *r*. One can observe that when the total layer number is beyond 12, the results of multilayer beams are very closed to the results of monolithic ones, implying that a multilayer GPLRC beam with 12 or more layers is an excellent approximation for an ideal functionally graded beam structure with a continuous and smooth variation in both material composition and properties. In addition, this result also demonstrates that the new FG-GPLRC structures proposed in present work can be realized easily using the multilayer alternatives in the manufacturing processing.

## 6. Conclusions

A new distribution law based on the error function has been proposed and is further employed to introduce a novel FG-GPLRC. The GPL distributions in the thickness direction can be adjusted by a gradient index to maintain a constant total GPL weight fraction. The modified Halpin–Tsai micromechanics model is used to evaluate the effective Young’s modulus of the FG-GPLRCs.

A computational modeling based on an improved third-order shear deformation theory and Chebyshev–Ritz methodology is developed. The comparisons prove that the developed modeling is more accurate and efficient. The bending deflections, stresses, and natural frequencies of a novel FG-GPLRC beam are investigated using the proposed modeling. The parameter studies are performed, and some conclusions are summarized based the numerical results:

(1) With respect to the GPL distribution patterns, low-content GPL additions can significantly improve the stiffness and bending resistance of the beam.
(2) For the thick FG-GPLRC beam, the new distribution law can produce the stiffest FG-GPLRC beams with the lowest bending displacements and highest fundamental frequencies. In other words, by adjusting the gradient index, the most optimized distribution law for the new FG-GPLRCs can be found, which results in an FG-GPLRC beam with the greatest bending resistance and vibration stiffness.
(3) For the thin FG-GPLRC beam, the increase in gradient index reduces the bending displacements and increases the fundamental frequencies. In addition, if the gradient index is beyond 1.6, the new FG-GPLRCs exhibit better capabilities in the static and vibration analysis than the common FG-X ones.
(4) A multilayer GPLRC beam with 12 or more layers is an ideal alternative structure for fabricating the new FG-GPLRC structures.

## Figures and Tables

**Table 1:** Values of the indices for various boundary conditions.

Boundary Conditions	*L*_u_	*L*_ϕ_	*L_w_*	*R*_u_	*R*_ϕ_	*R_w_*
H-H	1	0	1	1	0	1
C-C	1	1	2	1	1	2
C-H	1	1	2	1	0	1

**Table 2:** Deflection $\bar{w}$, normal stress ${\bar{\sigma }}_{xx}$, shear stress ${\bar{\sigma }}_{xz}$ and natural frequency of FG sandwich beam.

*N*	$\bar{w}$	${\bar{\sigma }}_{xx}(0.5L,0.5h)$	${\bar{\sigma }}_{xz}(0,0)$	*ω*_1_	*ω*_2_	*ω*_3_
2	0.2665	0.7793	0.8495	4.6677	28.1358	35.2954
3	0.2664	0.7783	0.9104	4.6672	18.2622	27.9520
4	0.3281	1.1625	0.8476	4.2363	18.1846	27.9520
5	0.3281	1.1624	0.8448	4.2363	15.1507	27.9518
6	0.3281	1.1624	0.8416	4.2351	15.0990	27.9518
7	0.3281	1.1624	0.8416	4.2351	15.0990	27.9518
8	0.3281	1.1624	0.8416	4.2351	15.0990	29.5854
Ref. [59]	0.3282	1.1632	0.8371	4.3052		

**Table 3:** Comparisons of the vertical dimensionless mid-span displacements of functionally graded (FG) sandwich beams.

Source	H-H			C-C		
	*p* = 1	*p* = 5	*p* = 10	*p* = 1	*p* = 5	*p* = 10
FSDT [60]	5.4408	8.1409	9.0232	1.3770	2.0635	2.2614
TSDT [60]	5.4122	8.5762	9.4800	1.3372	1.9896	2.1747
Qusi-3D [60]	5.3612	8.5137	9.4050	1.3077	1.9416	2.1211
Present	5.3822	8.5140	9.4049	1.3126	1.9637	2.1487

**Table 4:** Comparisons of the dimensionless fundamental frequencies of the FG sandwich beams.

Source	H-H			C-C		
	*p* = 1	*p* = 2	*p* = 5	*p* = 1	*p* = 2	*p* = 5
HSDT [61]	4.1105	3.7334	3.3771	8.3747	7.7149	7.0723
TSDT [61]	4.1105	3.7334	3.3771	8.3705	7.7114	7.0691
Qusi-3D [61]	4.1185	3.7410	3.3840	8.4653	7.8008	7.1550
Present	4.1146	3.7374	3.3791	8.4163	7.7731	7.1265

**Table 5:** Maximum dimensionless displacements of the functionally graded graphene nanoplatelet reinforced composite (FG-GPLRC) beams (H-H).

*L*/*h*	Gradient Index	0%	0.25%	0.5%	0.75%	1.0%	1.25%	1.5%
5	*uniform*	15.4247	8.0196	5.4191	4.0927	3.2882	2.7483	2.3609
	*r* = 2	15.4247	6.4531	4.1506	3.0742	2.4460	2.0329	1.7402
	*r* = 4	15.4247	5.7356	3.7519	2.8576	2.3350	1.9865	1.7351
	*r* = 6	15.4247	5.4902	3.6468	2.8394	2.3727	2.0618	1.8364
	*r* = 8	15.4247	5.3602	3.5876	2.8274	2.3940	2.1081	1.9019
	*r* = 10	15.4247	5.2786	3.5469	2.8146	2.4019	2.1323	1.9394
20	*uniform*	13.9187	7.2366	4.8901	3.6931	2.9672	2.4800	2.1304
	*r* = 2	13.9187	5.4633	3.4046	2.4744	1.9442	1.6015	1.3618
	*r* = 4	13.9187	4.5728	2.7528	1.9752	1.5429	1.2673	1.0760
	*r* = 6	13.9187	4.2864	2.5560	1.8303	1.4302	1.1764	1.0007
	*r* = 8	13.9187	4.1479	2.4628	1.7627	1.3785	1.1355	0.9676
	*r* = 10	13.9187	4.0666	2.4087	1.7238	1.3490	1.1124	0.9492

**Table 6:** Maximum dimensionless displacements of the FG-GPLRC beams (C-C).

*L*/*h*	Gradient Index	0%	0.25%	0.5%	0.75%	1.0%	1.25%	1.5%
5	*uniform*	4.2718	2.2210	1.5008	1.1335	0.9107	0.7611	0.6538
	*r* = 2	4.2718	2.0630	1.4099	1.0798	0.8776	0.7402	0.6404
	*r* = 4	4.2718	2.0489	1.5206	1.2491	1.0734	0.9466	0.8494
	*r* = 6	4.2718	2.0297	1.5695	1.3427	1.1963	1.0891	1.0048
	*r* = 8	4.2718	2.0089	1.5836	1.3834	1.2575	1.1663	1.0947
	*r* = 10	4.2718	1.9912	1.5853	1.4011	1.2884	1.2083	1.1461
20	*uniform*	2.8595	1.4867	1.0046	0.7587	0.6096	0.5095	0.4377
	*r* = 2	2.8595	1.1424	0.7184	0.5250	0.4140	0.3419	0.2913
	*r* = 4	2.8595	0.9728	0.6006	0.4392	0.3482	0.2894	0.2482
	*r* = 6	2.8595	0.9175	0.5657	0.4165	0.3332	0.2795	0.2419
	*r* = 8	2.8595	0.8902	0.5488	0.4057	0.3264	0.2757	0.2402
	*r* = 10	2.8595	0.8739	0.5386	0.3992	0.3224	0.2734	0.2393

**Table 7:** Maximum dimensionless displacements of the FG-GPLRC beams (C-H).

*L*/*h*	Gradient Index	0%	0.25%	0.5%	0.75%	1.0%	1.25%	1.5%
5	*uniform*	7.5135	3.9064	2.6397	1.9936	1.6017	1.3387	1.1500
	*r* = 2	7.5135	3.4008	2.2651	1.7109	1.3784	1.1557	0.9956
	*r* = 4	7.5135	3.2236	2.2761	1.8171	1.5328	1.3344	1.1859
	*r* = 6	7.5135	3.1492	2.2960	1.8972	1.6517	1.4789	1.3474
	*r* = 8	7.5135	3.1002	2.2946	1.9303	1.7105	1.5572	1.4409
	*r* = 10	7.5135	3.0651	2.2863	1.9427	1.7390	1.5990	1.4938
20	*uniform*	5.8586	3.0460	2.0583	1.5545	1.2489	1.0439	0.8967
	*r* = 2	5.8586	2.3178	1.4503	1.0567	0.8317	0.6859	0.5837
	*r* = 4	5.8586	1.9552	1.1908	0.8621	0.6781	0.5602	0.4779
	*r* = 6	5.8586	1.8379	1.1131	0.8076	0.6382	0.5300	0.4546
	*r* = 8	5.8586	1.7807	1.0759	0.7820	0.6200	0.5170	0.4454
	*r* = 10	5.8586	1.7468	1.0541	0.7670	0.6095	0.5095	0.4403

**Table 8:** Dimensionless fundamental frequencies of the FG-GPLRC beams (H-H).

*L*/*h*	Gradient Index	0%	0.25%	0.5%	0.75%	1.0%	1.25%	1.5%
5	*uniform*	2.7510	3.8158	4.6427	5.3432	5.9621	6.5225	7.0386
	*r* = 2	2.7510	4.2651	5.3256	6.1935	6.9478	7.6247	8.2444
	*r* = 4	2.7510	4.5350	5.6241	6.4572	7.1538	7.7643	8.3151
	*r* = 6	2.7510	4.6392	5.7135	6.4913	7.1141	7.6421	8.1065
	*r* = 8	2.7510	4.6969	5.7644	6.5112	7.0902	7.5672	7.9764
	*r* = 10	2.7510	4.7340	5.7995	6.5292	7.0827	7.5291	7.9046
20	*uniform*	2.9268	4.0597	4.9394	5.6846	6.3430	6.9393	7.4884
	*r* = 2	2.9268	4.6725	5.9202	6.9457	7.8372	8.6366	9.3677
	*r* = 4	2.9268	5.1077	6.5850	7.7759	8.8002	9.7123	10.5423
	*r* = 6	2.9268	5.2758	6.8343	8.0789	9.1418	10.0828	10.9349
	*r* = 8	2.9268	5.3632	6.9627	8.2328	9.3125	10.2640	11.1221
	*r* = 10	2.9268	5.4166	7.0405	8.3256	9.4143	10.3708	11.2306

**Table 9:** Dimensionless fundamental frequencies of the FG-GPLRC beams (C-C).

*L*/*h*	Gradient Index	0%	0.25%	0.5%	0.75%	1.0%	1.25%	1.5%
5	*uniform*	5.3197	7.3788	8.9778	10.3324	11.5291	12.6129	13.6109
	*r* = 2	5.3197	7.6810	9.3025	10.6367	11.8043	12.8582	13.8276
	*r* = 4	5.3197	7.7226	8.9804	9.9178	10.7058	11.4055	12.0454
	*r* = 6	5.3197	7.7644	8.8480	9.5769	10.1536	10.6478	11.0904
	*r* = 8	5.3197	7.8076	8.8135	9.4412	9.9106	10.2972	10.6339
	*r* = 10	5.3197	7.8441	8.8118	9.3854	9.7956	10.1215	10.3976
20	*uniform*	6.5386	9.0696	11.0349	12.6999	14.1709	15.5030	16.7297
	*r* = 2	6.5386	10.3472	13.0511	15.2697	17.1980	18.9272	20.5089
	*r* = 4	6.5386	11.2140	14.2769	16.6999	18.7603	20.5820	22.2321
	*r* = 6	6.5386	11.5474	14.7112	17.1514	19.1832	20.9484	22.5245
	*r* = 8	6.5386	11.7233	14.9375	17.3787	19.3812	21.0964	22.6079
	*r* = 10	6.5386	11.8322	15.0780	17.5201	19.5036	21.1854	22.6525

**Table 10:** Dimensionless fundamental frequencies of the FG-GPLRC beams (C-H).

*L*/*h*	Gradient Index	0%	0.25%	0.5%	0.75%	1.0%	1.25%	1.5%
5	*uniform*	3.9767	5.5159	6.7112	7.7238	8.6184	9.4286	10.1746
	*r* = 2	3.9767	5.9341	7.2835	8.3886	9.3517	10.2182	11.0134
	*r* = 4	3.9767	6.1126	7.2968	8.1804	8.9168	9.5648	10.1527
	*r* = 6	3.9767	6.1901	7.2756	8.0200	8.6064	9.1038	9.5442
	*r* = 8	3.9767	6.2417	7.2826	7.9571	8.4646	8.8798	9.2376
	*r* = 10	3.9767	6.2788	7.2983	7.9352	8.3986	8.7669	9.0768
20	*uniform*	4.5406	6.2982	7.6630	8.8192	9.8407	10.7658	11.6176
	*r* = 2	4.5406	7.2203	9.1296	10.6976	12.0606	13.2829	14.4007
	*r* = 4	4.5406	7.8622	10.0781	11.8488	13.3635	14.7076	15.9278
	*r* = 6	4.5406	8.1096	10.4252	12.2445	13.7795	15.1260	16.3366
	*r* = 8	4.5406	8.2391	10.6045	12.4444	13.9819	15.3181	16.5093
	*r* = 10	4.5406	8.3185	10.7144	12.5662	14.1037	15.4314	16.6070

**Table 11:** Maximum displacements ${\bar{w}}_{\max}$ and fundamental frequencies Ω_1_ of multilayer beams.

Total Layer Number	${\bar{w}}_{\max}$			Ω_1_		
	*r* = 1	*r* = 3	*r* = 5	*r* = 1	*r* = 3	*r* = 5
2	0.8810	1.8431	4.1067	11.9650	8.2686	5.5381
4	0.8633	0.9409	1.3221	12.0850	11.5581	9.7511
8	0.8639	0.9674	1.1190	12.0809	11.3910	10.5805
12	0.8643	0.9720	1.1323	12.0778	11.3637	10.5198
16	0.8645	0.9743	1.1368	12.0764	11.3504	10.4995
20	0.8646	0.9775	1.1425	12.0748	11.3324	10.4871
50	0.8648	0.9777	1.1442	12.0746	11.3304	10.4656
Monolithic	0.8648	0.9777	1.1452	12.0746	11.3302	10.4610
